# Computational Construction of Toxicant Signaling Networks

**DOI:** 10.1021/acs.chemrestox.2c00422

**Published:** 2023-07-20

**Authors:** Jeffrey N. Law, Sophia M. Orbach, Bronson R. Weston, Peter A. Steele, Padmavathy Rajagopalan, T. M. Murali

**Affiliations:** † Interdisciplinary Ph.D. Program in Genetics, Bioinformatics, and Computational Biology, Blacksburg, Virginia 24061, United States; § Department of Chemical Engineering, Virginia Tech, Blacksburg, Virginia 24061, United States; ∥ Department of Computer Science, Virginia Tech, Blacksburg, Virginia 24061, United States

## Abstract

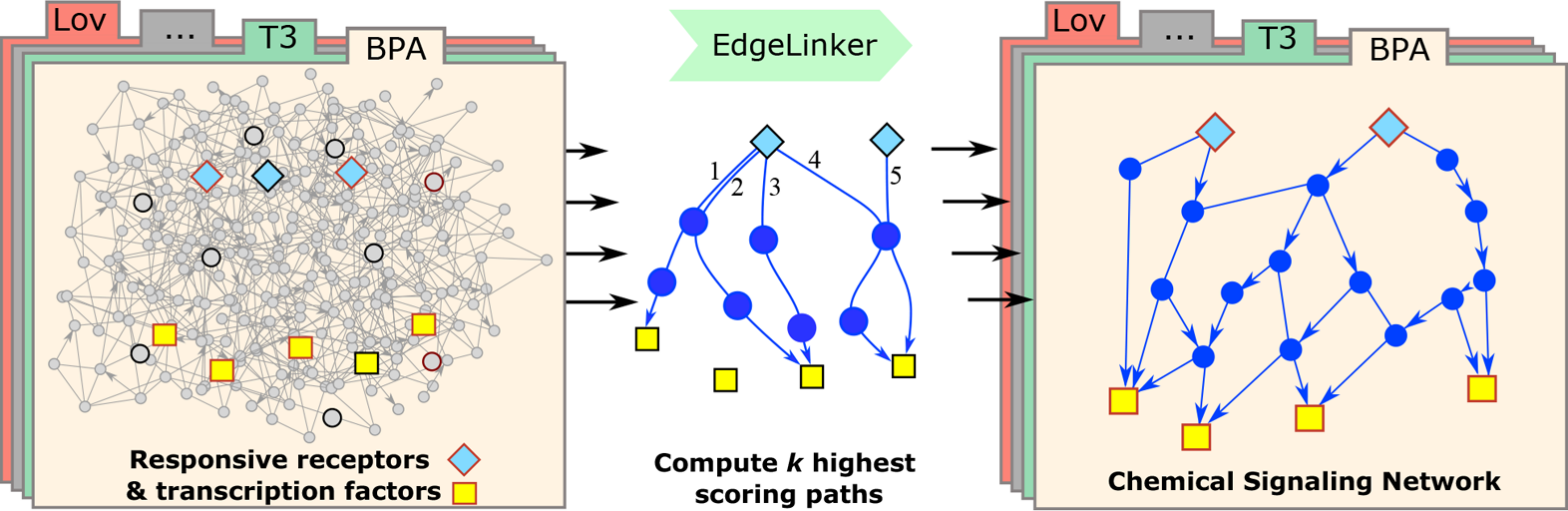

Humans and animals are regularly exposed to compounds that may have adverse effects on health. The Toxicity Forecaster (ToxCast) program was developed to use high throughput screening assays to quickly screen chemicals by measuring their effects on many biological end points. Many of these assays test for effects on cellular receptors and transcription factors (TFs), under the assumption that a toxicant may perturb normal signaling pathways in the cell. We hypothesized that we could reconstruct the intermediate proteins in these pathways that may be directly or indirectly affected by the toxicant, potentially revealing important physiological processes not yet tested for many chemicals. We integrate data from ToxCast with a human protein interactome to build toxicant signaling networks that contain physical and signaling protein interactions that may be affected as a result of toxicant exposure. To build these networks, we developed the EdgeLinker algorithm, which efficiently finds short paths in the interactome that connect the receptors to TFs for each toxicant. We performed multiple evaluations and found evidence suggesting that these signaling networks capture biologically relevant effects of toxicants. To aid in dissemination and interpretation, interactive visualizations of these networks are available at http://graphspace.org.

## 1 Introduction

The United States Environmental Protection Agency (EPA) has identified approximately 85,000 chemicals with partially or entirely unknown effects on humans and animals.^24^ Investigating the potential toxicity of each of these chemicals through traditional animal testing would require an insurmountable investment of time and resources and cause extensive loss of animal life. Therefore, the EPA established the Toxicity Forecaster (ToxCast) program to prioritize chemicals for in-depth toxicity testing.^13^ ToxCast, and its extension Tox21, have collected data on over 9,000 chemicals including pesticides, industrial chemicals, and pharmaceuticals.^1^ Over 1,000 high-throughput assays have been used to investigate a range of biological responses induced by each of these chemicals. ToxCast utilizes both cell-based and cell-free assays to analyze changes to proteins, enzymatic activity, phenotype, and secreted factors such as cytokines and chemokines in response to chemical treatment.^28,48^

The publicly available ToxCast data are an excellent resource for determining how a chemical is biotransformed and which proteins it interacts with. This data has improved our understanding of potential chemical hazards and their mechanisms of action.^17,25,28,37,48^ The ToxCast assays are performed independently of each other and most often measure the response of a single protein. However, proteins do not act in isolation. Rather, they interact via complex networks to perform specific tasks such as signal transduction, metabolism, and motility. Integrating the ToxCast data with molecular interaction networks, which represent physical binding or signaling between proteins, chemicals, and other molecules, could improve our understanding of the effects of a chemical on cellular pathways. This type of integration has been applied in other closely related contexts, such as analyzing and predicting molecular perturbations caused by drugs^6,34,45^ and pesticides.^3,30^ To the best of our knowledge, no approach has combined the ToxCast data with human protein interactions to identify potential signaling pathways affected by a chemical.

**Figure 1:**
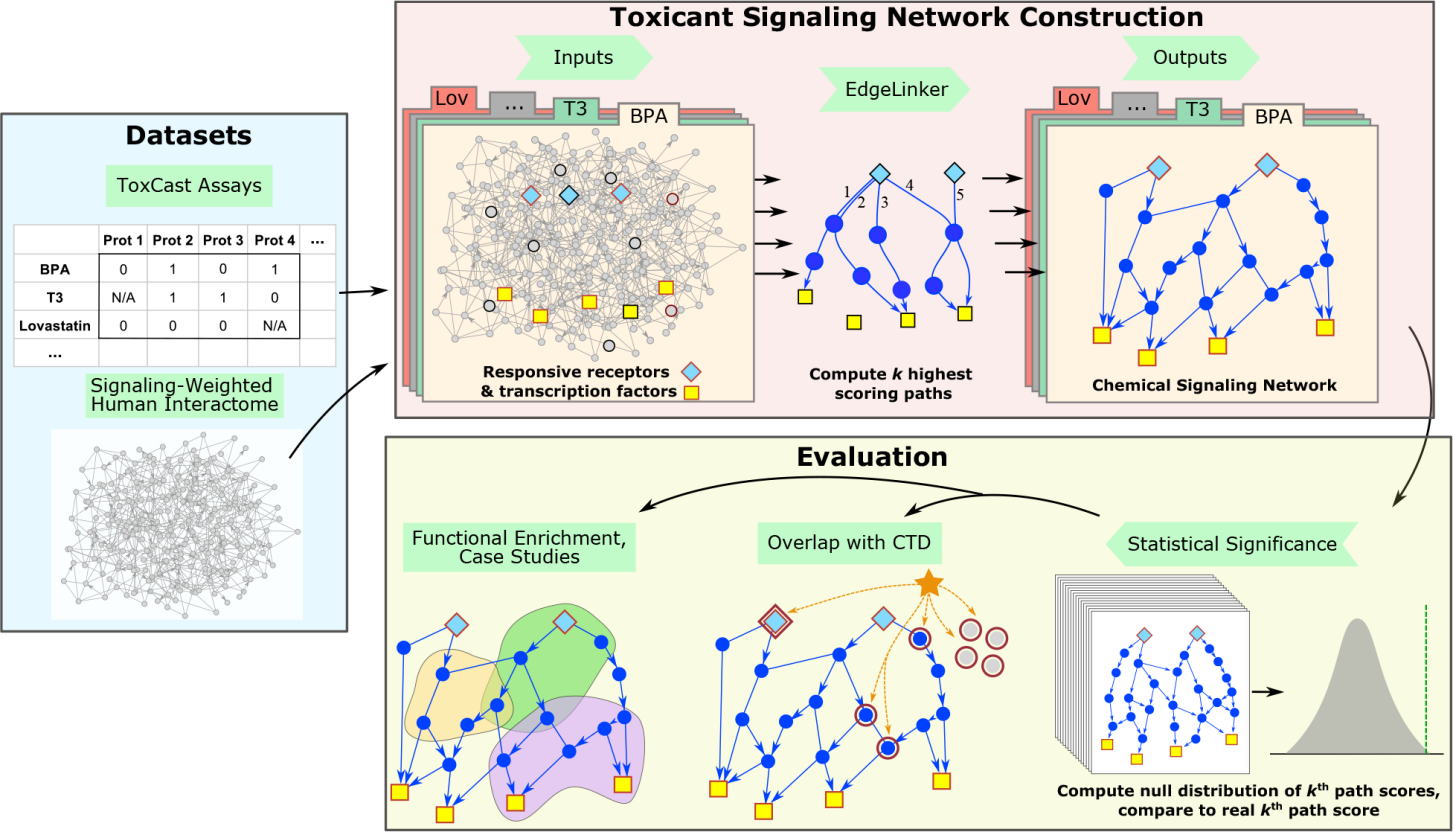
Overview of the analysis framework. The toxicant signaling networks were constructed using the results of ToxCast assays and a human protein interaction network as inputs. The networks were evaluated by statistical analysis and validated using functional enrichment, the Comparative Toxicogenomics Database (CTD), and literature-based investigation of bisphenol A (BPA) and lovastatin (Lov).

In this work, which is an extension of a Ph.D. study,^31^ we focus on integrating the ToxCast data with a human protein interaction network to build *toxicant signaling networks*, which we define as the signaling and physical protein interactions that may be affected by toxicant exposure. These toxicant signaling networks may reveal important intermediate proteins and physiological processes not yet tested for many chemicals. For each chemical, we identified the affected receptors and transcription factors (TFs) as determined by ToxCast assays. Subsequently, we sought to connect these receptors (sources or causes) to the TFs (targets or effects) through the human protein interaction network (Figure 1).

One state-of-the-art method called PathLinker reconstructs signaling pathways by computing multiple short paths from receptors to the TFs of a pathway within an interactome.^43^ In an evaluation against many other algorithms, PathLinker demonstrated the highest accuracy in reconstructing known signaling pathways.^43^ Herein, we developed a modified version of PathLinker called EdgeLinker, which is better suited for our goal of computing toxicant signaling networks. Based on these networks, we computed signal transduction pathways and other biological functions that may be affected by each toxicant. Finally, we conducted a detailed literature review of two chemicals to validate the relevance of the signaling networks on known cellular perturbations.

## 2 Materials and Methods

### 2.1 Toxicant Signaling Networks

We start with a directed, weighted network *G* = (*V*, *E*, *w*) where each node *u* ∈ *V* is a human protein, and each edge (*u*, *v*) ∈ *E* represents a physical interaction between two proteins. The weight *w*(*u*, *v*) of such an edge is based on the amount of evidence (e.g., yeast 2-hybrid, phosphorylation assays) supporting the corresponding interaction. Each edge weight will lie between 0 and 1. The larger the weight, the greater the amount of evidence for the edge. We provide more details on how we construct this network in Section 2.4. For a given toxicant, *x*, we define *S*_*x*_ and *T*_*x*_ to be the receptors (sources) and TFs (targets), respectively, reported to be responsive to *x* in the ToxCast data (Section 2.5) that are also members of *V*. We define a toxicant signaling networkfor *x* to be the subnetwork within *G* connecting the receptors in *S*_*x*_ to the TFs in *T*_*x*_, as computed by the algorithm we describe next.

We defined the following criteria for an algorithm that computes toxicant signaling networks:

- 1. To reflect the process of signal transduction, the responsive receptors *S*_*x*_ must be connected to the downstream responsive TFs *T*_*x*_ in the output network. Specifically, for each receptor in *S*_*x*_, there must be a path in the toxicant signaling networkto some TF in *T*_*x*_ and vice versa.
- 2. The algorithm must have a tunable parameter to control the size of the subnetwork.
- 3. Increasing the size parameter should ensure that at least one edge is added to the subnetwork.

PathLinker, which computes the *k* highest scoring, simple paths from any source in *S*_*x*_ to any target in *T*_*x*_, is well suited for this task, except it fulfills only the first two requirements. The reason is that increasing *k*, i.e., the number of paths from *S*_*x*_ to *T*_*x*_, does not always add new edges to the computed subnetwork, since every edge in a new path may have occurred in an earlier path.

### 2.2 The EdgeLinker Algorithm

Motivated by the criteria outlined above and our observations on PathLinker, we developed a new algorithm for computing toxicant signaling networks, which we call EdgeLinker. For every edge (*u*, *v*) ∈ *E*, the objective of EdgeLinker is to identify the shortest path from any node in *S*_*x*_ to any node in *T*_*x*_ that utilizes that edge. If edge weights are present, EdgeLinker finds the lowest cost path, where the cost of a path is the sum of the costs of its edges, and we define the cost of an edge to be the negative of the logarithm of its weight. Note that the lowest cost path is also the highest scoring path, where the score of a path is the product of the weight of each edge in it. This computation will yield |*E*| or fewer paths, since two different edges may share a lowest cost path from *S*_*x*_ to *T*_*x*_. Subsequently, given an integer parameter *k* > 0, EdgeLinker outputs the *k* paths with the highest score. The network formed by these paths is the desired toxicant signaling network. EdgeLinker uses the following steps:

- 1. Add a super source *s* to *V*. For every receptor *u* in *S*_*x*_, add a directed edge (*s*, *u*) with a weight of one to *E*. Note that each of these edges will have a cost of zero after taking the logarithm of its weight.
- 2. Add a super target *t* to *V*. For every node *u* in *T*_*x*_, add a directed edge (*u*, *t*) with a weight of one.
- 3. Run Dijkstra’s algorithm^12^ to compute the shortest path in *G* from *s* to every node in *V*. Note that the second node in every such path must be a receptor in *S*_*x*_.
- 4. Create the graph *G*′ = (*V*, *E*′). For every edge (*u*, *v*) in *E*, add the edge (*v*, *u*) to *E*′ with the same weight. Thus, the nodes of *G* and *G*′ are identical whereas each edge in *G*′ is the reverse of the corresponding edge in *G*.
- 5. Run Dijkstra’s algorithm to compute the shortest path in *G*′ from *t* to every node in *V*.
- 6. Convert every path π computed in the previous step into a path in *G* by reversing the edges in π. Note that the penultimate node in every such path must be a TF in *T*_*x*_. This computation gives us the shortest path from every node in *V* to *t*.
- 7. For each edge (*u*, *v*) in *G*, to compute the shortest path from *s* to *t* through (*u*, *v*), concatenate the shortest path from *s* to *u*, the edge (*u*, *v*) and the shortest path from *v* to *t*.
- 8. Rank these paths in increasing order of cost and delete the nodes *s* and *t* from each path.

We ran EdgeLinker for each toxicant. To select the value for the number of paths cutoff *k*, we sought to balance the trade-off between too many paths that would overwhelm interpretation and likely include incorrect predictions (i.e., false positives) and too small a subnetwork that would likely miss important signals (i.e., false negatives). We performed a few different analyses to guide our selection of *k* (Section 3, Supporting Information Section 2) and set *k* = 150. Ultimately, we provide all the computed paths and the edges ranked by the first path in which they appear as supplementary files (see the github link in the Data Availability Statement); thus, a smaller or larger cutoff for *k* can easily be applied if so desired.

### 2.3 Statistical Significance Test for Signaling Networks

We sought to develop a statistical significance test to answer the following question: if we randomly swapped interaction partners between all proteins in the background interactome, would we find as many short paths connecting the responsive receptors and TFs as were computed using the original interactome? We devised a two-step strategy to assess significance.

First, we generated 10,000 “randomized” background interactomes by sampling uniformly at random from the distribution of degree-preserved, edge-swapped networks (i.e., interactomes) using the following method: two edges *a*, *b* and *c*, *d* are selected at random from the network and replaced with the two swapped edges *a*, *d* and *b*, *c* with two constraints: (1) no duplicate edges and (2) no self-loops were allowed. This method ensures that the degree of each node is preserved, but the interacting partners are randomly assigned. We recognize that our interactome contains both directed and undirected edges and that treating them all as directed or undirected in the randomized networks would lead to an unfair or biased comparison to the original interactome. Thus, we swapped the directed and undirected edges separately from each other while maintaining the two above constraints.

Second, for each chemical *c*, we generated a null set of signaling networks (*Y*) to compare to the original signaling network by running EdgeLinker on each of the 10,000 randomized interactomes using the same receptors and TFs responsive to *c* as the sources and targets. To compare the signaling network of *c* with its corresponding null networks, we computed *s*_*k*_, the path score (the product of the individual edge weights on the path) at the *k*th (i.e., 150th) path and counted the number of paths with that score or higher. We then estimated a permutation *p*-value for each toxicant signaling network by computing the fraction of random networks with a greater number of paths at that score (*s*_*k*_) than the chemical’s signaling network using the equation
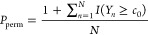
where *c*_0_ is the number of paths with a score ≥ *s*_*k*_ in the original network for *c*, *Y*_*n*_ is the number of paths in the random network *n* with a score ≥ *s*_*k*_, *I*(*Y*_*n*_ ≥ *c*_0_) is 1 if *Y*_*n*_ is ≥ *c*_0_ and 0 otherwise, and *N* is the total number of random networks tested (i.e., 10,000). The pseudocount is added because if we could enumerate all possible random networks, at least one would be identical with the original interactome, causing the minimum *p*-valueto be 1/*N*. We chose to compare the number of paths at *s*_*k*_ rather than the score of the *k*th path, because in some cases there can be a large number of ties.

The null hypothesis of this test is that the number of paths connecting a set of sources and targets above a path score cutoff is dependent on the degree of each node in the interactome and not on the specific connections between them. A small *p*-value *P*_perm_ (e.g., < 0.01) would indicate that we reject the null hypothesis and accept the alternative hypothesis that the number of paths connecting a set of sources and targets is dependent on the specific interactions in the interactome. The smaller the *p*-value *P*_perm_, the lower the number of random networks that are connected as good or better than the signaling network.

Since the resolution of *P*_perm_ is limited by our testing range (i.e., 1/*N*) and correcting for multiple hypothesis testing could push most networks over the cutoff (e.g., 1/10000 × 389 = 0.039), we sought to estimate more accurate *p*-values. Knijnenberg et al.^29^ showed that the generalized Pareto distribution (GPD), which approximates the distribution of the extreme values of a set of independent and identically distributed random variables, can be used to fit the tail of the distribution *c*_*n*_ to better approximate the true *p*-value (i.e., if *N* covered all permutations). They utilized maximum likelihood estimation to fit the two GPD parameters *a*, the scale parameter, and *k*, the shape parameter, with the 250 most extreme permutation values and then employed a goodness-of-fit test to assess whether the exceedances follow a GPD. If the goodness-of-fit test fails, then the number of extreme permutation values is decreased by 10 until the test passes. If the test never passes, then this approach should not be used. We did not have any such cases in this work.

We used the python implementation available at https://github.com/jlaw9/gpdPerm. To correct for multiple hypothesis testing, we computed a *q*-value using the Benjamini-Hochberg correction.^4^

### 2.4 Human Interactome

We combined protein–protein interactions (PPIs) available from physical molecular interaction databases (BioGrid, DIP, InnateDB, IntAct, MINT, and PhosphositePlus)^11,16^ and signaling pathway databases (NetPath and SPIKE),^27,40^ downloaded in January 2018. We mapped all of the identifiers to UniProtKB accession numbers. Since only 4% of edges were directed signaling interactions from these databases, we also utilized the orientations predicted by Silverbush and Sharan.^47^ For each predicted direction, *u* → *v* or *v* → *u* that overlapped with an undirected interaction (*u*, *v*) from the aforementioned PPI databases, we replaced (*u*, *v*) with the corresponding directed edge. The resulting network contained 16,713 nodes, 93,179 regulatory (directed) interactions, and 205,404 physical (undirected) interactions, where multiple types of evidence supported many of the edges. We next converted each undirected edge (*u*, *v*) to two directed edges *u* → *v* and *v* → *u* resulting in 503,987 edges. We provide this interactome as well as all of the evidence supporting each edge as supplementary files (see the github link in the Data Availability Statement).

We assigned a confidence *w*_*uv*_ ∈ (0, 1] to each directed edge (*u*, *v*) ∈ *E* that (*u*, *v*) is a true interaction based on the amount of evidence supporting (*u*, *v*).^54^ This method assigns a confidence score to each experiment type/pathway database and then combines those scores into a weight for each edge using a Bayesian approach. For a more detailed explanation, see the Supporting Information.

To prioritize directed over undirected edges in our toxicant signaling networks, we artificially decreased the weights of undirected edges by applying a penalty. In addition, since the ToxCast assays report the level of perturbation by the chemical (Section 2.5), we implemented a strategy for EdgeLinker to prioritize paths through proteins in *S*_*x*_ and *T*_*x*_ that are more strongly affected by *x*. See the Supporting Information for more details.

### 2.5 Processing the ToxCast Data

We selected the chemicals for this study using the ToxCast version 3 data from the file *hitc_Matrix_190708.csv* downloaded from the ToxCast Web site^1^ in August 2019. This file lists 9,224 chemicals that have been studied by ToxCast and reports the activity of 1,473 different high throughput screening assays. The assays correspond to hundreds of proteins including receptors and TFs. This file also reports functional assays including apoptosis, cell cycle, and proliferation in response to these chemicals. There are four possible recorded values for each chemical-assay pair:

- 1. 1 (hit): AC_50_ ≤ 50 μM, i.e., the estimated concentration of the chemical that perturbed the assay activity by at least 50% was at most 50 μM;
- 2. 0 (nonhit): AC_50_ > 50 μM;
- 3. −1: Fewer than four concentrations of the chemical were tested or the activity could not be determined;
- 4. NA: The chemical was not tested for that assay

To facilitate the integration of the ToxCast data with protein interaction networks, we mapped each protein assayed by ToxCast to its corresponding UniProtKB accession number. Of the original 1,092 assays, 801 assays corresponded to a human protein UniProtKB accession number. These assays spanned 298 unique human proteins. We deemed a protein to be “responsive” to a chemical if the corresponding assay had a value of “1” in the hit matrix. If multiple ToxCast assays corresponded to the same protein, we deemed a protein to be responsive to a chemical if all of these assays had a value of 1 in the hit matrix, except for up/down assay pairs, which we treat as a single assay. We also retrieved a measurement of the severity of the effect of the chemical on an assay using the reported *z*-score in the file *zscore_Matrix_190708.csv*. If multiple assays tested a single protein, we took the maximum of the corresponding *z*-scores.

We narrowed down the 9,224 chemicals to the 1,387 chemicals that were successfully tested (0 or 1 reported) for at least 500 assays. We restricted our attention to 505 chemicals that had at least one responsive human receptor and one responsive human TF. We determined whether an assayed protein was a receptor or a TF from the *intended_target_type_sub* column of the “Assay Summary” file, provided the *intended_target_family* was not “nuclear receptor”. In addition, we limited the receptor assays to those made by Novascreen (NVS) since they tested for direct receptor-chemical interactions. A total of 52 receptors and 42 TFs assayed fit this criteria.

### 2.6 Signaling Evidence in CTD

We downloaded the chemical–gene interactions from the Comparative Toxicogenomics Database (CTD)^10^ in March 2020 and limited the data to human genes. Although CTD does not contain a true set of gold standard edges or nodes for a toxicant signaling network against which we can evaluate our results, it does provide information about which proteins are involved in a phosphorylation or dephosphorylation reaction as a result of toxicant exposure. Since cellular responses to chemicals often use these reactions, we used this information in CTD as a proxy gold standard. We limited the CTD chemical–gene interactions to those with the word “phosphorylation” or “dephosphorylation” in the interaction column, which gives us a set of genes for each chemical. We then mapped the proteins in our toxicant signaling networksto gene names and performed a hypergeometric test to calculate the statistical significance of the overlap between the two sets.

### 2.7 Functional Enrichment Using DAVID and Revigo

For each toxicant signaling network, we tested for enrichment of Gene Ontology (GO) Biological Process (BP) terms among the proteins of the network using the DAVID web service.^18^ Specifically, we used the “GOTERM_BP_DIRECT” category with the default settings and applied a Bonferroni-corrected *p*-value cutoff of 0.01.

To summarize the resulting list of terms, we used Revigo^50^ which aims to remove redundancy by clustering terms based on semantic similarity and selecting a representative term from each cluster based on the enrichment *p*-value. We used the default settings, except for setting the similarity threshold to 0.5 (i.e., “Small”), and using “Homo sapiens” for the GO term sizes database.

For each chemical, we also computed the enrichment of terms among all of the proteins labeled as responsive (i.e., 1 in the hit matrix) using DAVID as outlined above.

## 3 Results

**Figure 2:**
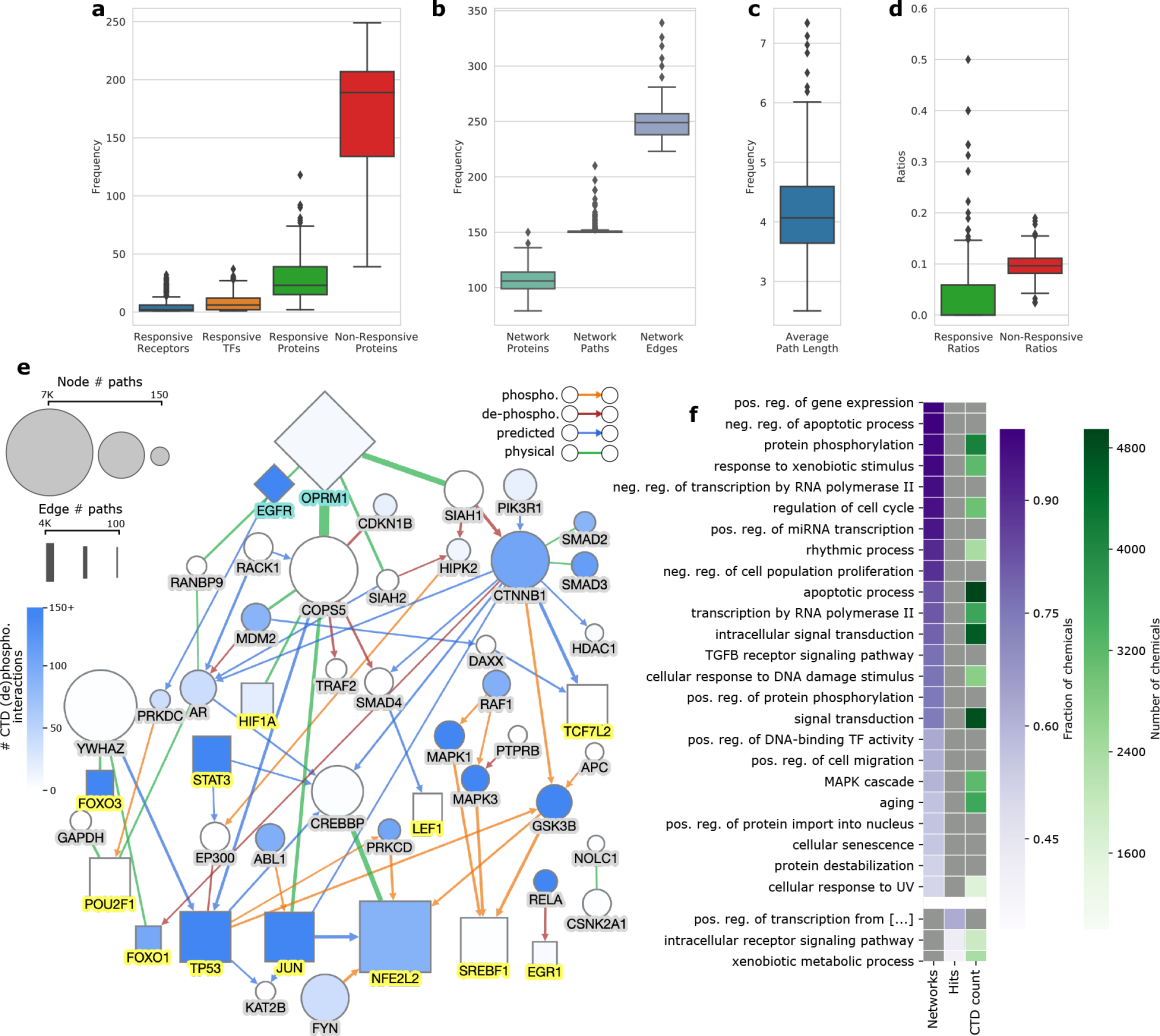
(a) Distribution of the number of responsive receptors and transcription factors (TFs) from the ToxCast data for each chemical and the number of responsive and nonresponsive proteins from the ToxCast data set. (b) Distribution of the number of proteins, paths, and edges in each toxicant signaling network. (c) Distribution of the average path length of each toxicant signaling network. (d) Distribution of the ratio of intermediate responsive and nonresponsive proteins recalled in the toxicant signaling networksto the original ToxCast data for each chemical. The boxes contain the middle 50% of the data with the lines representative of the medians. Outliers are marked by diamonds and were determined by assessing data outside 1.5× interquartile range from the median. (e) Overview of the nodes and edges present in the 198 statistically significant toxicant signaling networks (i.e., *q*-value <0.01) where an edge was included if it was present in a third or more of the networks. The node and edge sizes correspond to the total number of paths they participated in across the 198 networks, and the intensity of blue among nodes indicates the number of (de)phosphorylation interactions from CTD in which a given gene was involved. Diamonds: receptors assayed in ToxCast, squares: TFs assayed in ToxCast. Edge colors indicate the following: orange: phosphorylation, maroon: dephosphorylation, blue: predicted direction, green: physical interaction (Section 2.4). (f) Fraction of chemicals for which a term is enriched in the signaling network (left) or responsive proteins (middle). The CTD counts column (green) indicates the number of chemicals in CTD for which that function is enriched.

We built a signaling network for each of the 389 chemicals with at least one receptor and TF that were responsive in ToxCast assays by using EdgeLinker to compute the *k* = 150 shortest paths from the responsive receptors (sources) to the responsive TFs (targets) through the human interactome (Section 2.2). These chemicals perturbed a median of two receptors and six TFs (distributions in Figure 2a). The resulting networks contained a median of 106 proteins and 249 edges (Figure 2b), 75% of which were regulatory interactions (i.e., directed edges). The distribution of the average path length (i.e., number of edges) per toxicant signaling networkis shown in Figure 2c, where the average length across all networks was 4.1. We also computed the number of edges along the shortest receptor-to-TF paths in every signaling pathway in the NetPath and KEGG databases. These paths had an average of 3.1 (SD = 1.2) and 4.2 (SD = 2.3) edges, respectively, indicating that for toxicant signaling networks we computed, the average path length fell within the range of curated signaling pathways.

Since there were many other assays in ToxCast, we measured the fraction of proteins that were responsive and nonresponsive to each toxicant that were also present in its signaling network, excluding the receptors and TFs used as inputs to EdgeLinker. We expected these fractions to be small since these assays tested cell-free, direct perturbations of the protein by a chemical rather than assessing indirect perturbation by cellular processes, such as signal transduction. Indeed, the median fraction of responsive and nonresponsive proteins was 0.0 and 0.1 (Figure 2d), suggesting there was no relationship between the intracellular proteins directly perturbed by a toxicant in a cell-free assay and the proteins found on signaling paths from perturbed receptors to TFs.

An important factor to consider is that TF assays could be responsive to a toxicant for a variety of different reasons (see the Discussion section). In addition, many human TFs and receptors were not tested meaning a perturbed receptor could have carried a signal to an untested TF and vice versa. We devised a statistical significance test to help identify which toxicant signaling networks are more likely to have TFs activated or deactivated by signals carried from perturbed receptors. We hypothesized that cellular signals carried from receptors to TFs would have evolved to be more efficient than using random interactions to pass signals. Therefore, if, for a given chemical, the lengths of the shortest paths between its responsive receptors and TFs were shorter than would be expected by chance, we assume that the paths in the computed network are more likely to carry real cellular signals. Based on this hypothesis, we developed a test for the statistical significance of a toxicant signaling network (Section 2.3). A total of 198 out of 389 networks achieved a *q*-value <0.01. We conducted all further analyses on these 198 significant networks.

### 3.1 Overlap of Toxicant Signaling Networks with CTD

The first evaluation we performed of the 198 statistically significant toxicant signaling networks was to check a different source for support of the proteins in our networks. While no gold standard database of toxicant signaling networks currently exists, the Comparative Toxicogenomics Database (CTD)^10^ contains chemical–gene/protein interactions manually curated from the literature. These interactions include direct effects such as physical binding and indirect effects such as changes in gene expression or the phosphorylation of a protein due to cellular exposure to the chemical. Since our goal was to capture the signaling effects of each toxicant and phosphorylation is the primary mechanism of cellular signaling, we focused our attention on toxicant–protein interactions where the protein was phosphorylated or dephosphorylated.

We first performed a high-level analysis by investigating whether CTD contained evidence for the (de)phosphorylation of proteins common to many of the signaling networks due to chemical exposure. We observed that of the 49 proteins found in a third or more of our networks, 34 of them had at least some evidence of signaling in CTD (blue nodes in Figure 2e). These findings support the idea that proteins commonly found in our toxicant signaling networksare also involved in signaling-type interactions for many chemicals.

Next, we checked whether evidence of proteins being (de)phosphorylated due to chemical exposure in CTD overlapped with the proteins in each toxicant signaling network. Of the 198 toxicants, only 26 of them had at least one protein with evidence of signaling in CTD. For many of those chemicals, the lack of evidence is most likely because the majority of chemicals in the ToxCast data set are not well studied. For example, only 116 of them have any kind of chemical–gene interaction in the CTD, with 43 having 10 or more. Of those 26 toxicants, 20 had a statistically significant amount of proteins with evidence of (de)phosphorylation (hypergeometric test *q*-value <0.05). Cumulatively, this provides strong support to our hypothesis that the toxicant signaling networkscapture biologically relevant pathways connecting chemically perturbed receptors to chemically sensitive TFs.

### 3.2 Functional Enrichment

We sought to understand the cellular functions of the proteins in each of the toxicant signaling networks. In addition, since the ToxCast assays tested many other proteins than the receptor and TF assays we used to build the signaling networks (e.g., nuclear receptors and intracellular enzymes), we wanted to compare how the functions among the ToxCast-reported responsive proteins (i.e., hits) differ from those in the networks we built. We tested for enrichment of Gene Ontology (GO) biological processes terms among the proteins in each signaling network, as well as among the hits of each toxicant, using DAVID^18^ (Section 2.7). Since the networks were all built from a limited set of receptors and TFs (51 and 42, respectively), we expected there to be some overlap of the enriched functions among the 198 statistically significant signaling networks. We first counted the number of times each term was enriched across the signaling networks and found 44 functions present in 50% or more of them. Since there were many somewhat redundant terms among this list, we used Revigo^50^ to summarize them, which reduced the number of terms to 24. For the responsive proteins, only one term was common to 50% or more of the chemicals, so we relaxed the threshold to 33% which included two more terms.

We observed that these functions fell into two groups: those enriched among the proteins in the toxicant signaling networks and those enriched among the responsive proteins (Figure 2f). Many of the terms common among the networks were related to regulation (e.g., “negative regulation of apoptotic process“) and to signaling (e.g., “transforming growth factor beta signaling pathway”), whereas the three terms in the second group were more general terms (e.g., “xenobiotic metabolic process”). Thus, we get a high-level view of the complementary nature of the networks to the ToxCast data. Some of the functions common among the toxicant signaling networks were somewhat surprising including “regulation of cell cycle”, “cellular response to DNA damage stimulus”, “protein destabilization”. These terms may indicate more general cellular stressors induced by many of these toxicants.

To get a sense for if the nongeneral functions (i.e., annotation frequency <5%) among these two groups are affected by chemicals in general, we compared to the functions enriched among genes affected by each chemical in CTD (not restricted to those tested by ToxCast). We observed that 11 of the 21 nongeneral terms among the two groups were also enriched for 1,000 or more chemicals in CTD (green column of Figure 2f), supporting the idea that these functions are commonly affected by a wide variety of chemicals.

### 3.3 Case Studies

In order to examine the success of the EdgeLinker algorithm at identifying biologically meaningful pathways, we further investigated the signaling networks of two well studied chemicals relevant to human toxicity: lovastatin and bisphenol A (BPA). For each chemical, we tested for functional enrichment among the proteins in its signaling network using DAVID and summarized those terms using Revigo (Section 2.7). We then analyzed and compared those proteins and corresponding functions to the ToxCast responsive proteins and known perturbed biological functions.

#### 3.3.1 Lovastatin

**Figure 3:**
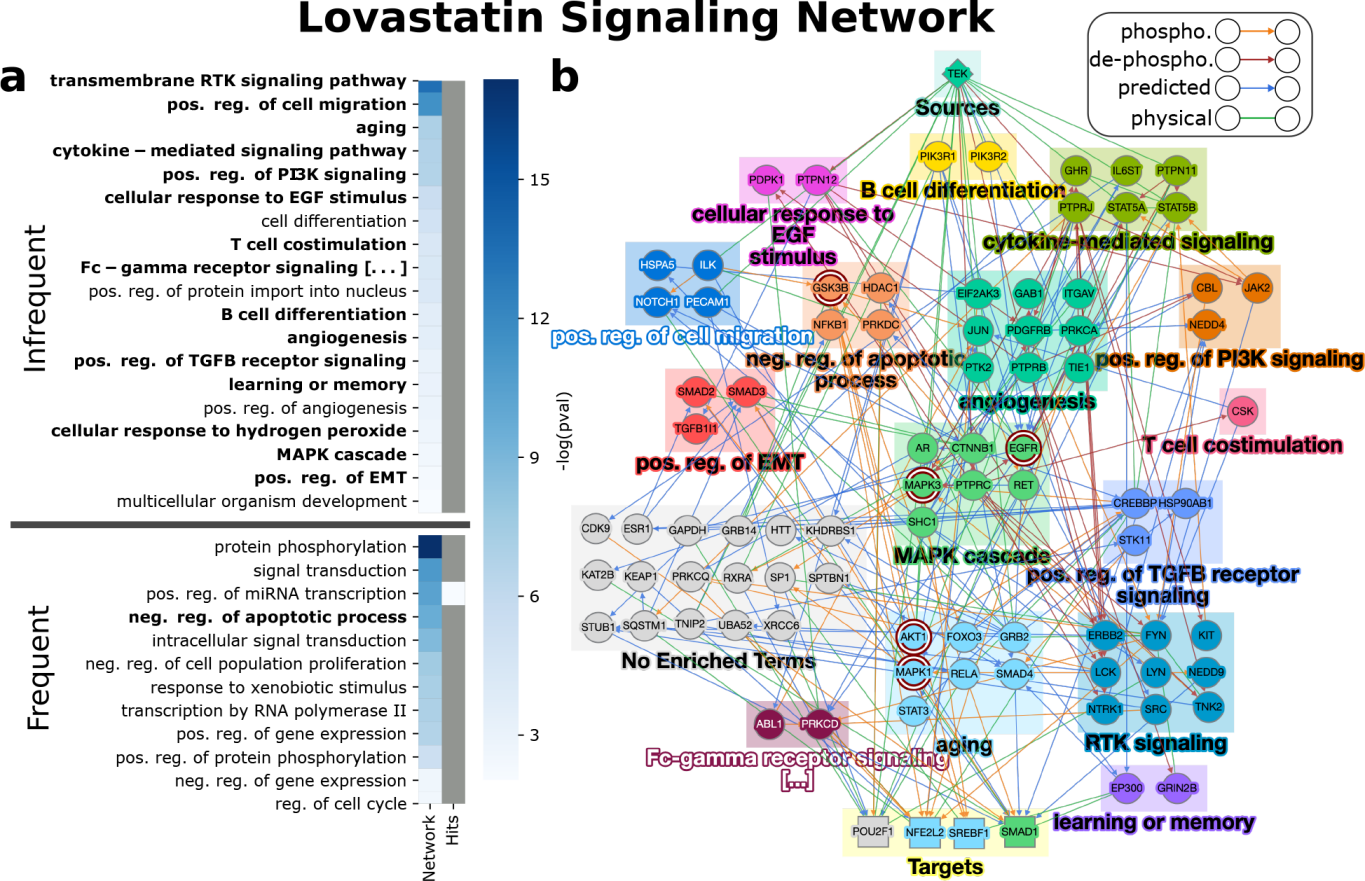
Lovastatin enriched functions and signaling network. (a) GO BP terms enriched among the proteins of the signaling network and all ToxCast-reported hits for lovastatin. Terms are split into two groups: Frequent (frequency among signaling networks ≥75%), and infrequent (frequency among signaling networks <75%). Terms are in bold if they were selected for b. (b) Toxicant signaling networkwhere nodes are colored by the first term in which they are enriched according to the order in a. Nodes with a maroon border have signaling evidence in CTD.

Lovastatin, a cholesterol-lowering drug, was tested in 750 human ToxCast assays corresponding to 107 proteins. Of those tested, one receptor (TEK), five TFs (MTF1, NFE2L2, POU2F1, SMAD1, and SREBF1), and eight intermediate proteins were classified as responsive. EdgeLinker established a network of 150 paths with 86 proteins and 231 interactions (Figure 3b, *q*-value = 5.8 × 10^–9^). We first checked if CTD contained evidence of signaling. Of the 86 proteins, 5 overlapped with the 10 proteins involved in phosphorylation in CTD for lovastatin (nodes with maroon double border in Figure 3b; hypergeometric test *q*-value= 4.4 × 10^–9^). DAVID computed 65 functions enriched among these 86 proteins (available in supplementary files (see the github link in the Data Availability Statement)). We used Revigo (Section 2.7) to summarize these functions, which reduced the number of GO terms to 31 (Figure 3a). Using the low-dimensional representation of terms computed by Revigo to select a representative term for small groups of functions, we further manually filtered this list to 16 terms to highlight (bold terms and corresponding colored boxes in Figure 3).

Importantly, we found literature support connecting almost all of the functions enriched in the signaling network for lovastatin to observed cellular effects of the chemical, as well as many of the proteins in the network. In addition to reducing cholesterol, statins have an anti-inflammatory effect through many factors including by suppressing the expression of pro-inflammatory cytokines and chemokines.^36^ The first terms we discuss are related to these anti-inflammatory effect, namely “cytokine-mediated signaling pathway”, “positive regulation of cell migration”, “T cell costimulation”, and “JAK-STAT cascade involved in growth hormone signaling pathway” (enriched, but not selected by Revigo). Cheng et al. tested the effects of lovastatin on human T cells and found that it dose-dependently inhibited stimuli-induced cytokine production such as IL2, IL4, and INF-gamma.^7^ Jougasaki et al. showed that statins reduce the inflammation of human vascular endothelial cells by suppressing monocyte migration through inhibition of the JAK/STAT pathway.^23^ They and others demonstrated that statins inhibit the phosphorylation of several JAK and STAT proteins,^46^ including JAK2 and STAT3 which are present in the lovastatin signaling network. Lovastatin has also been shown to reduce brain inflammation in mice by inhibiting leukocyte migration.^15^ Paths in the signaling network provide hypotheses for mechanisms by which lovastatin may inhibit inflammation through (paths through genes in the green “cytokine-mediated signaling” rectangle in the top-right corner Figure 3b), positive regulation of cell migration (blue square in top-left corner), and the JAK/STAT pathway (e.g., TEK → PTPN11 → STAT3 and TEK → PTPN12 → JAK2).

Lovastatin also has a beneficial antitumor effect by inhibiting several other signaling pathways. These include the transmembrane RTK, MAPK, EGF, TGFβ, and insulin receptor signaling pathways,^8,14,20,32,38,53^ all of which are enriched in the signaling network for lovastatin (“transmembrane RTK signaling pathway”, “MAPK cascade”, “cellular response to EGF stimulus”, “positive regulation of TGFβ receptor signaling pathway”, and “insulin receptor signaling pathway”).

We next discuss several terms related to toxicity. A negative side-effect of lovastatin is thrombocytopenia which is low blood platelet count. In mice, lovastatin significantly reduced circulating platelets by inducing platelet apoptosis.^55^ The signaling network for lovastatin may explain the mechanisms by which this occurs through inhibition of proteins involved in the “negative regulation of apoptotic process” and “angiogenesis” terms (orange, green, and red squares near top-center of Figure 3b), as well as “positive regulation of blood vessel endothelial cell migration” (enriched but not selected).

Finally, we discuss the terms “aging” and “learning or memory”. Statin therapy has been shown to reduce the mortality rate of patients between the age 70–90, independent of blood cholesterol levels.^21^ Using *C. elegans* as a model for aging, Jahn et al. found that lovastatin prolonged lifespan through activation of JNK1 and DAF-16/FOXO3a,^22^ whose human homologues MAPK8 and FOXO3 are present in our signaling network. For “learning or memory”, lovastatin has been shown to alleviate cognitive deficits of Noonan syndrome in mice by inhibiting the ERK signaling pathway in the brain (“positive regulation of ERK1 and ERK2 cascade” also enriched but not selected by Revigo). In a small clinical trial, lovastatin had beneficial effects on some learning and memory functions for patients with Neurofibromatosis Type 1.

The only enriched term for which we could not find a direct link in the literature to lovastatin was “ephrin receptor signaling pathway” (not selected by Revigo). Ephrins signal via EphAs and EphBs, which mediate a variety of essential processes in human embryonic development such as axon guidance and cell migration and in adults they play a critical role in tissue homeostasis and in the immune system.^9,33^ While this connection could be a false positive, the effect of lovastatin on members of the Eph signaling pathway may be a novel link to help explain lovastatin’s anti-inflammatory and antitumor effects.

#### 3.3.2 Bisphenol A (BPA)

BPA is commonly used in industry in the production of many products, most prominently polycarbonates and epoxy resins.^19,49^ The chemical is an endocrine disrupter associated with a host of health effects including reproductive issues, birth defects, carcinogenesis, and metabolic diseases such as type II diabetes and obesity.^44^

**Figure 4:**
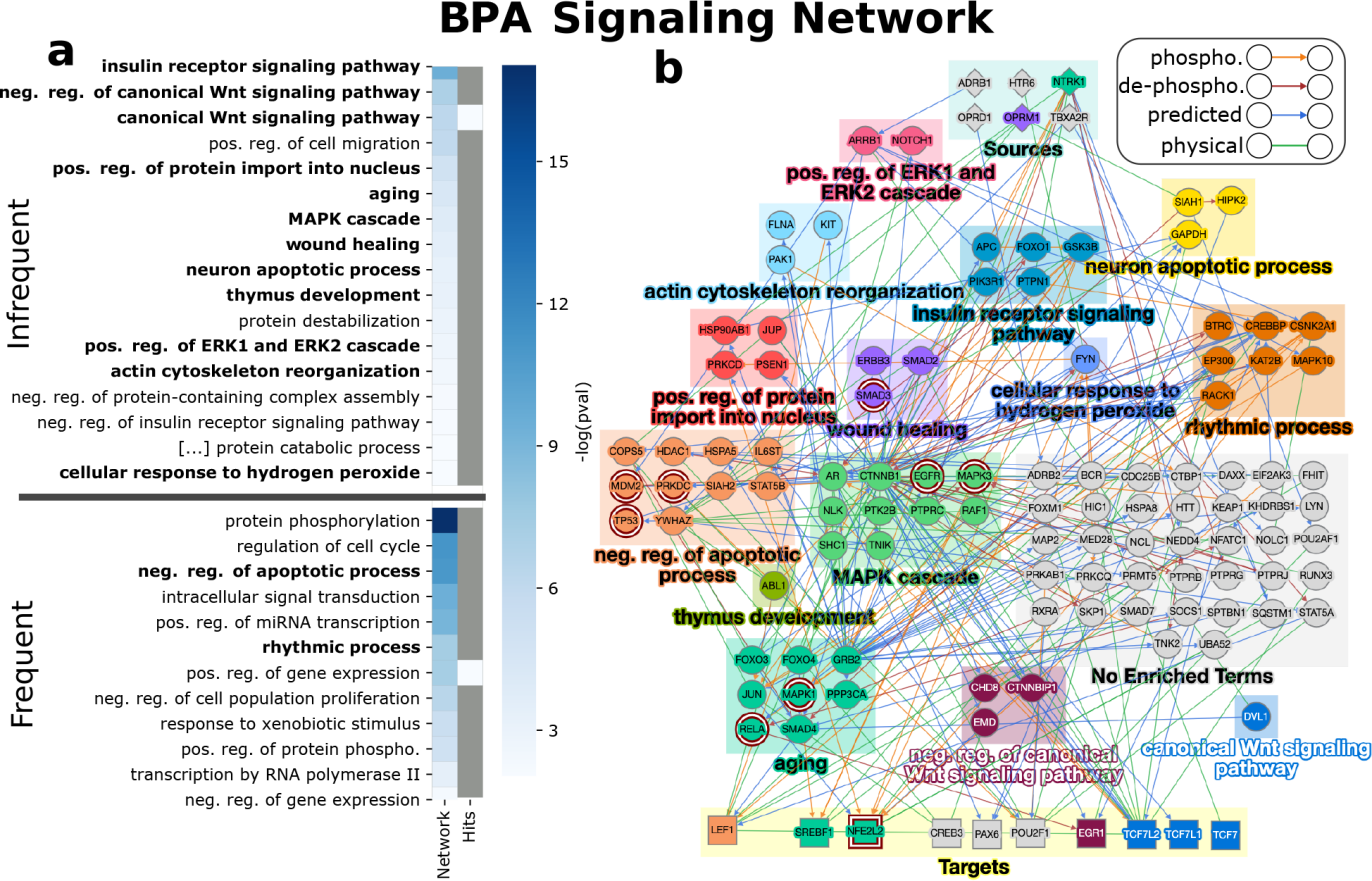
BPA enriched functions and signaling network. (a) GO BP terms enriched among the proteins of the network and all ToxCast-reported hits for BPA. Terms are split into two groups: Frequent (frequency among signaling networks ≥75%), and Infrequent (frequency among signaling networks <75%). Terms are bold if they were selected for b. (b) Toxicant signaling networkwhere nodes are colored by the first term in which they are enriched according to the order in a. Nodes with a maroon border have signaling evidence in CTD.

BPA was tested on 989 human ToxCast assays corresponding to 152 proteins. Of those tested, 10 receptors, 11 TFs, and 27 intermediate proteins were responsive. EdgeLinker established a signaling network with 114 proteins and 248 interactions (Figure 4, *q*-value of 2.3 × 10^–3^). We first examined the CTD for evidence of phosphorylation. Of the 114 proteins in the network, 9 overlapped with the 30 proteins involved in phosphorylation in CTD for BPA (nodes with maroon double border in Figure 4b; hypergeometric test *q*-value = 2.8 × 10^–12^), which was one of the largest amount of overlaps among the signaling networks. GO term enrichment analysis using DAVID resulted in 50 enriched functions, which were reduced to 29 by Revigo. From these 29 terms, we selected 14. The variation in these functions is representative of the myriad health effects associated with BPA-induced toxicity.

Similar to lovastatin, we found literature support for the effect of BPA on the functions enriched in its signaling network. We first discuss the relation of BPA’s carcinogenic potential with the terms “MAPK cascade”, “positive regulation of ERK1 and ERK2 cascade”, and “negative regulation of the apoptotic process”. BPA induced activation of MAPK and ERK1/2 pathways in human ovarian cancer cells, promoting proliferation.^41^ In human colon adenocarcinoma cells, BPA significantly promoted proliferation in a time- and dose-dependent manner via activation of the ERK pathway.^26^ In addition, BPA has been linked to increased apoptosis in leukemia cell lines.^5^

We also discuss the connections of functions enriched in the signaling network for BPA (Figure 4b) with their effects on developmental neurotoxicity. Prenatal exposure to BPA has been directly associated with differences in children’s brain microstructure.^39^ Neurotoxicity has been shown through an increase in reactive nitrogen and oxygen species in human glutamatergic neurons^52^ and in rats^2^ which could be because of activation of proteins involved in the “cellular response to hydrogen peroxide” (FYN in blue square near middle of Figure 4b as well as ABL1, FOXO1, MDM2, NFE2L2, and RELA) and “regulation of nitric oxide biosynthetic process” (EGFR, ESR1, SMAD3, and others in Figure 4b). Inhibition of the WNT/β-catenin signaling pathway in rat embryos also led to nerotoxicity^35,51^ (“negative regulation of canonical Wnt signaling pathway” and “canonical Wnt signaling pathway”, maroon and blue squares at bottom of Figure 4b). The proteins annotated to the term “neuron apoptotic process” (yellow square, top-right of Figure 4b, as well as TP53, HSPA5, and PSEN1, among others)) may provide additional mechanisms of the developmental neurotoxic effects of BPA.

We note that the estrogen receptor signaling pathway and similar terms are not enriched in the signaling network for BPA. This may be because the estrogen receptors tested by ToxCast are nuclear receptors, which we did not consider in our analysis since they can act as TFs by directly binding to DNA. The G-protein-coupled estrogen receptor 1 (GPER1) was not tested by ToxCast. Nevertheless, many of the nonestrogenic effects of BPA are captured by the signaling network.

## 4 Discussion

Little is known about many of the potential effects on human and environmental health of many of the chemicals currently used in industry. The ToxCast program seeks to quickly and efficiently evaluate the potential toxicity of thousands of chemicals and prioritize them for further testing.^42^ We sought to further expand the utility of these data and the resources available to toxicologists by combining the interactome of human protein–protein interactions with the ToxCast receptor and TF assays to compute toxicant signaling networks. These networks differ from other frameworks for toxicant exposure such as adverse outcome pathways (AOPs) in that AOPs aim to capture the many different molecular and cellular events caused by exposure to a chemical that induces a toxic effect in an organism. The aim of the toxicant signaling networks in this work is to identify potential signaling pathways and other cellular functions affected by a toxicant. These networks serve as another resource to aid toxicologists in understanding the molecular effects of these toxicants.

We note some limitations of these signaling networks due to the ToxCast assays on which they rely. Due to the nature of the cellular TF assays, it can be difficult to interpret the cause of an assay reporting a perturbation (i.e., hit). In some cases, changes in TF activity would be due to a perturbed membrane-bound receptor. These were the ideal mechanistic actions we hoped to uncover using the computed toxicant signaling networks. However, it would also be possible for a chemical to diffuse into a cell and perturb the TF directly or affect a nuclear receptor or other intracellular enzyme which could lead to changes in TF activity. Furthermore, a chemical could be overly promiscuous in the number of affected assays through general cytotoxicity.^42^ These types of dynamics would likely be missed in our methodology. Further work and likely additional experimental assays would be necessary to disentangle these different processes.

To assess the quality of these signaling networks, we performed multiple evaluations, including a comparison of the length of paths in each network to those from randomized interactomes, an analysis of potential supporting evidence in CTD, and a thorough literature evaluation for two well-studied chemicals. We found that the signaling networks can, in fact, be correlated with the cellular responses to chemicals in the body. The functions, specific proteins, and interactions in the signaling networks provide potential hypotheses of signaling mechanisms by which the chemicals affect the cell, which could be especially useful for less studied chemicals.

## Data Availability

The toxicant signaling networks, as well as the data and software necessary to create them and the other results, are available at https://github.com/Murali-group/tox_signaling_networks. This includes the interactome, the paths computed by EdgeLinker for each toxicant, the enriched GO terms, as well as several supporting files such as the evidence for each interaction in the interactome. We also provide a snapshot of the repository at https://doi.org/10.5281/zenodo.8147563. Each of the toxicant signaling networks, as well as the network in Figure 2e, are available for visualization and download on GraphSpace at http://graphspace.org/graphs/?query=tags:2023-toxicant-signaling-networks.

## Supporting Information Available

Describes weighting the interactome and the effect of the number of paths (*k*) cutoff (PDF)
