# Supplementary material for: Computational Construction of Toxicant Signaling Networks
Source: Chem Res Toxicol. 2023 Jul 20;36(8):1267–77. doi: 10.1021/acs.chemrestox.2c00422 (PMC10445288; doi:10.1021/acs.chemrestox.2c00422)
Supplement: Supplementary file 1 — tx2c00422_si_001.pdf [file tx2c00422_si_001.pdf]

— Supplementary Information —
Computational Construction of Toxicant Signaling Networks

Jeffrey N. Law[1,2], Sophia M. Orbach[3], Bronson R. Weston[1], Peter A. Steele[4], Padmavathy Rajagopalan[3], and T. M. Murali[4,*]

[1]Interdisciplinary Ph.D. Program in Genetics, Bioinformatics, and Computational Biology, Blacksburg, VA 24061, USA
[2]Present address: Biosciences Center, National Renewable Energy Laboratory, 15013 Denver West Parkway Golden, CO 80401, USA
[3]Department of Chemical Engineering, Virginia Tech, Blacksburg, VA 24061, USA
[4]Department of Computer Science, Virginia Tech, Blacksburg, VA 24061, USA
[*]Corresponding author: murali@cs.vt.edu

June 7, 2023

Contents

1 Weighting the human interactome **S2**

2 Effect of number of paths (k) cutoff **S3**

1 Weighting the human interactome

To assign a confidence score to each edge in the interactome, we started with the same probabilistic approach used by Poirel *et al.*[1]. Given a pair of proteins u and v, let $I \in 0, 1$ be a binary random variable such that $I = 1$ if u and v truly interact, and $I = 0$ otherwise. Let $E = [E_1, \ldots, E_n] \in 0, 1^n$ be a vector of binary random variables, where $E_k = 1$ if evidence type i (e.g., yeast 2-hybrid) supports an interaction between u and v, and $E_k = 0$ otherwise. We compute w_{uv} given the experimental evidence for the interaction between u and v as

$$\begin{aligned} w_{uv} &= \Pr(I = 1|E) \\ &= \frac{\Pr(E|I = 1)\Pr(I = 1)}{\Pr(E)} \qquad (1) \\ &= \frac{\Pr(E|I = 1)\Pr(I = 1)}{\Pr(E, I = 0) + \Pr(E, I = 1)} \\ &= \frac{\Pr(I = 1)\Pi_k \Pr(E_k|I = 1)}{\Pr(I = 0)\Pi_k \Pr(E_k|I = 0) + \Pr(I = 1)\Pi_k \Pr(E_k|I = 1)}, \qquad (2) \end{aligned}$$

where Equation (1) is an application of Bayes rule and Equation (2) assumes conditional independence of the evidence types conditioned on I such that $\Pr(E|I) = \Pi_k \Pr(E_k|I)$.

Let P and N be disjoint sets of positive and negative protein pairs, respectively. We used the GO terms "cell surface receptor signaling pathway" (GO:0007166) and "cellular response to chemical stimulus" (GO:0070887) to select proteins that are related to signaling and/or response to a chemical for defining P and N. We labeled each protein pair (u, v) as a positive example if u and v were co-annotated with either of these terms. For N, we sampled $10 \times |P|$ protein pairs uniformly at random that were not co-annotated to these functions. We set the prior probability of an interaction $P(I)$ to $\Pr(I = 1) = \frac{|P|}{|P \cup N|}$ and $\Pr(I = 0) = \frac{|N|}{|P \cup N|}$.

Let X_k be the set of protein pairs observed to interact under evidence type k (i.e., the set of edges in the interactome with evidence type k). We computed the probability of an evidence type E_k conditioned on I as

$$\Pr(E_k = e|I = i) = \begin{cases} \frac{P \cap X_k}{P}, & \text{if } e = 1, i = 1 \\ \frac{N \cap X_k}{N}, & \text{if } e = 1, i = 0 \\ \frac{P \setminus X_k}{P}, & \text{if } e = 0, i = 1 \\ \frac{N \setminus X_k}{N}, & \text{if } e = 0, i = 0 \end{cases}$$

We calculated the confidence of each evidence type k as $Pr(I = 1|E)$ where $E_k = 1$ and $E_j = 0$ for evidence type $j \neq k$. We include the confidence scores for each evidence type with more than 100 interactions as a supplementary file.

Since EdgeLinker computes minimum cost paths, we transformed the edge probabilities to costs (where smaller is better) by taking the absolute value of the log of the weight. To mitigate the influence of the highest-weighted edges, we applied a penalty to all edges by dividing each edge weight by 1.5 (in other words, adding the $\log_2 1.5$ to each edge cost). This approach gives edges with the highest weight (i.e., 0.99) a cost of 0.415 which is about half the cost of an edge with a weight of 0.66 (cost: 0.82). To prioritize directed edges over undirected edges, we applied an additional penalty to undirected edges by dividing their weights by 1.25, which gives undirected edges with the highest weight (i.e., 0.99) a cost of 0.64.

In addition, since each ToxCast assay reports a z-score which represents the level of assay perturbation by the chemical, we sought to use this information in our approach by prioritizing paths to/from receptors and TFs with higher z-scores. In order for these z-scores to be usable by EdgeLinker which finds minimum cost paths, we applied a penalty to each of the edges in G connected to the nodes in S and T as follows: We first found the maximum z-score z_{max} among all receptor and TF assays. Then, for each node $n \in S_x \cup T_x$ for a given toxicant t, we transformed the z-score corresponding to n $(z(n))$ to a penalty

$$p(n) = \begin{cases} 1 - (z(n)/z_{max}) & \text{if } n \in S_x \cup T_x \\ 0 & \text{otherwise} \end{cases}$$

such that higher z-scores have a smaller penalty, and then added $p(n)$ to the cost of each edge connected to n.

2 Effect of number of paths (k) cutoff

We sought to evaluate the effect of the cutoff on the number of paths (k) per signaling network on the statistical significance results, the overlap with CTD, and functional enrichment. We compared several values for k from $k = 25$ to $k = 500$. We found that the number of networks passing the significance threshold (q-value < 0.01) increased as k increased, from 90 for $k = 25$ to 225 for $k = 500$ (Table 1).

**k**	25	50	75	100	150	200	500
**# networks q-value < 0.01**	90	148	171	178	198	205	225

Table 1: Effect of the parameter k (number of paths) on the number of toxicant signaling networks with a q-value < 0.01.

For the analysis of the overlap of toxicant signaling network proteins with CTD (Section 3.1), we observed that larger k values increase the number of chemicals with a statistically significant overlap. For example, 18 chemicals had a q-value < 0.05 for $k = 100$, whereas for $k = 150$, that number increased to 20.

For functional enrichment, we noticed a similar trend: the presence of more proteins in the signaling networks led to an increase in the number of enriched terms. For example, for lovastatin, 42 and 65 terms were enriched (p-value < 0.01) for $k = 100$ and $k = 150$, respectively. For BPA, the number of terms increased from 40 to 50. We observed that these additional terms were typically quite similar to the smaller set.

To choose which value of k to apply, we computed, for a given value of k, the average number of edges in paths in a given toxicant signaling network. We then computed the mean of these values over all toxicants. We chose the largest value of k for which this (second) average path length fell within the expected range of path lengths among signaling pathways in NetPath and KEGG (3.1 – 4.2; see Results), which was $k = 150$ with an average path length equal to 4.1. Ultimately, as mentioned in section 2.2, a smaller or larger cutoff for k can be applied as desired since we provide all the computed paths and their ranks. On GraphSpace, a smaller k cutoff can be directly applied, allowing users to visually examine and "step-through" each path in a given toxicant signaling network.

References

[1] C. L. Poirel, R. R. Rodrigues, K. C. Chen, J. J. Tyson, and T. M. Murali. Top-down network analysis to drive bottom-up modeling of physiological processes. *Journal of Computational Biology*, 20(5):409–418, May 2013.
